# A database application for pre-processing, storage and comparison of mass spectra derived from patients and controls

**DOI:** 10.1186/1471-2105-7-403

**Published:** 2006-09-05

**Authors:** Mark K Titulaer, Ivar Siccama, Lennard J Dekker, Angelique LCT van Rijswijk, Ron MA Heeren, Peter A Sillevis Smitt, Theo M Luider

**Affiliations:** 1 Department of Neurology, Erasmus MC, Dr. Molewaterplein 40, P.O. Box 1738, 3000 DR Rotterdam, The Netherlands; 2 Department of Urology, Erasmus MC, Dr. Molewaterplein 40, P.O. Box 1738, 3000 DR Rotterdam, The Netherlands; 3 FOM-institute for Atomic and Molecular Physics, Kruislaan 407, 1098 SJ Amsterdam, The Netherlands

## Abstract

**Background:**

Statistical comparison of peptide profiles in biomarker discovery requires fast, user-friendly software for high throughput data analysis. Important features are flexibility in changing input variables and statistical analysis of peptides that are differentially expressed between patient and control groups. In addition, integration the mass spectrometry data with the results of other experiments, such as microarray analysis, and information from other databases requires a central storage of the profile matrix, where protein id's can be added to peptide masses of interest.

**Results:**

A new database application is presented, to detect and identify significantly differentially expressed peptides in peptide profiles obtained from body fluids of patient and control groups. The presented modular software is capable of central storage of mass spectra and results in fast analysis. The software architecture consists of 4 pillars, 1) a Graphical User Interface written in Java, 2) a MySQL database, which contains all metadata, such as experiment numbers and sample codes, 3) a FTP (File Transport Protocol) server to store all raw mass spectrometry files and processed data, and 4) the software package R, which is used for modular statistical calculations, such as the Wilcoxon-Mann-Whitney rank sum test. Statistic analysis by the Wilcoxon-Mann-Whitney test in R demonstrates that peptide-profiles of two patient groups 1) breast cancer patients with leptomeningeal metastases and 2) prostate cancer patients in end stage disease can be distinguished from those of control groups.

**Conclusion:**

The database application is capable to distinguish patient Matrix Assisted Laser Desorption Ionization (MALDI-TOF) peptide profiles from control groups using large size datasets. The modular architecture of the application makes it possible to adapt the application to handle also large sized data from MS/MS- and Fourier Transform Ion Cyclotron Resonance (FT-ICR) mass spectrometry experiments. It is expected that the higher resolution and mass accuracy of the FT-ICR mass spectrometry prevents the clustering of peaks of different peptides and allows the identification of differentially expressed proteins from the peptide profiles.

## Background

In mass spectrometry (MS), analysis of mass spectra is possible with various software packages. In general these software applications work fine for the analysis of individual spectra, but lack the ability to compare very large number of spectra and address differences in (peptide) profile masses to certain groups, such as patient and control groups. Therefore, it is necessary to have fast, user-friendly software for high throughput data pre processing, flexibility in changing input variables and statistical tools to analyze the peptides that are significantly differentially expressed between the patient and control groups. Statistical calculations are performed within seconds to at most several hours. To the best of our knowledge the only open source project that is capable of peptide profiling with raw MS fid (free induction decay) files (Bruker Daltonics, Germany) is the RProteomics 3-tier architecture of the Cancer Biomedical Informatics Grid, presented in a concurrent versions system (cabigcvs.nci.nih.gov). In the RProteomics project, the main development language is R and the application has a web interface. This paper describes an application where MS data preprocessing is expanded with a kind of Laboratory Information Management Systems (LIMS). It requires no grid architecture, can even be installed on a stand-alone computer, and due to local file interfaces can easily be integrated with commercial statistical software packages visualization applications, such as Spotfire™ [1] and Omniviz™. The presented software architecture is capable of central storage of mass spectra and analysis results. A central database holds all meta-data. Meta-data consist of the origin of the measured samples, experiments performed on different mass spectrometers and allocation of samples to different groups. Meta-data can also link the experimental results to clinical information. Information from the database can be retrieved with Structured Query Language (SQL) and can be linked to other databases on common keys, such as patient code. In this study, the application is built in fast Java code, which provides an excellent GUI, and statistic R routines are called if needed. In addition, the protein origin of the significant peptide masses can be identified by comparing the centrally stored peptide masses of interest with those calculated from the human mass spectrometry protein sequence database (for example MSDB) or by mass spectrometry assisted sequencing. Both identification techniques use the Mascot™ search engine [2]. The platform independent software architecture is tested on two sets of data: 1) Mass spectrometry (MS) files of cerebrospinal fluid (CSF) samples from patients with breast cancer, breast cancer with leptomeningeal metastasis (LM) and a control group [3]; and 2) MS files of serum samples from patients with prostate cancer in end stage disease and a control group.

## Implementation

### CSF samples of breast cancer patients

The processing of the CSF samples and measurement procedures have been described before [3,4]. In brief, each sample is processed twice, spotted 3 times on the anchor chip™ and measured three times on the mass spectrometer, which gives an average of 18 replicate spectra for each sample. Some measurements result in so called "zero" raw fid files with no data and a file size smaller than 5 Kbytes. This causes replicate numbers < 18. A (dataset dependent) replicate number of at least 7 spectra for each sample is proposed for robust statistic comparison between the groups [4]. The replicate number of 7 spectra for each sample is smaller than the total replicate number of 18, since some spectra cannot be internally calibrated on at least 4 of the 5 omnipresent albumin peptide masses. However, 7 replicate spectra are sufficient to find all possible peptide masses in all spectra [4]. A profile matrix is created, which consists of peptide masses in all spectra of the samples in the columns against the number of occurrences of these masses in replicate spectra of the samples in the rows. The matrix is created with the total number of 151 samples, control, breast cancer and breast cancer with LM and an arbitrary level of 450 peak masses for noise spectra, and peptide masses are selected between 800 and 4000 Da. The noise threshold of 450 peak masses in a spectrum can be varied in the database application. Peaks with masses smaller than 800 Da are left out because they may be attributed to matrix fragments. The replicate numbers of samples can be varied between 1 and 18. Table 1 gives an example of a quality report of the software, when a profile matrix is created. This quality report is automatically generated by the software architecture and stored as a text file, with an example of the name 0.98_1_ALBMASSES(4)_sample4_binary2_quality.txt, in the windows document root of the client workstation. In this name 98 represents the quantile percentage, 1 is the experiment number, ALBMASSES(4) is the name of the mass list, used for internal calibration, 4 in sample4 the number of chosen replicate spectra for one sample, which can be varied between 1 and 18 and 2 the threshold of the binary matrix table, as will be explained later. The windows documents root of the client workstation is C:\Documents and Settings\*username*\.

**Table 1:** Quality report. An example of a quality report, generated by the software, when a profile matrix is created.

Experimentid: 1 experimentname : cerebrospinal fluid tryptic peptide profiles to diagnose leptomeningeal metastasis in breast cancer patients Find maxima within distance (Da) : 0.50 Quantile threshold peak finding : 0.98 Combine peaks within distance (Da) : 0.50 Noisy spectra contain peak numbers > : 450 Maximum mass spectra per sample : 4 threshold binary table 0 < 1 > = : 2
Minimum mass (Da) : 800 Maximum mass (Da) : 4000 Calibration : yes ALBMASSES(4)

number of masses in matrix : 1949

group : control group without breast cancer(group1_control) total number of samples : 45 number of samples in matrix : 32 number of samples with too low number of replicates : 12 number of samples not in matrix that because all spectra cannot not be calibrated or are all too noisy : 1 (total number of 6 sample(s) with at least one noisy spectrum)

group : breast cancer without leptomeningeal metastasis(group2_breast_cancer) total number of samples : 52 number of samples in matrix : 39 number of samples with too low number of replicates : 13 number of samples not in matrix that because all spectra cannot not be calibrated or are all too noisy : 0 (total number of 5 sample(s) with at least one noisy spectrum)

group : breast cancer with leptomeningeal metastasis(group3_leptomeningeal_metastasis) total number of samples : 54 number of samples in matrix : 40 number of samples with too low number of replicates : 12 number of samples not in matrix that because all spectra cannot not be calibrated or are all too noisy : 2 (total number of 8 sample(s) with at least one noisy spectrum)

The profile matrix consists of peptide masses in all spectra of the samples on the columns against the number of occurrences of these masses in replicate spectra of the samples on the rows. The software stores the contents of the quality report as a text file, e.g. with the name 0.98_1_ALBMASSES(4)_sample4_binary2_quality.txt in the windows document root of the client workstation, namely in C:\Documents and Settings\*username*}\.

### Serum samples of prostate cancer patients

About 7 ml blood from 27 patients and 27 controls are collected in clotting tubes and stored at room temperature for a period of 2 h. Subsequently, the tubes are centrifuged at 1000 rpm for a period of 10 minutes, and the supernatant is collected and stored at a temperature of -80°C. The serum is tryptic digested and incubated overnight at a temperature of 37°C with a 1:10 ratio with a Promega Trypsin Gold stock solution, with a concentration of 100 μg/ml. In total 5 μl of the digested sample is bound to Magnetic Beads MB-HIC C18. The beads are eluted with 10 μl of 50% acetonitrile in Milli-Q water. An amount of 0.5 μl of the eluted fraction is spotted 4 times on the anchor chip and measured on an Ultraflex™ MALDI-TOF (Bruker Daltonics, Germany) in reflection mode, which gives 4 replicate spectra for each sample. The mass spectra internally calibrated on at least 4 of the 5 omnipresent peptide masses, which are different from those of the CSF experiment. A somewhat higher noise threshold than in the CSF samples is chosen of 600 peaks in a spectrum.

### Software architecture, packages and interfacing

**Figure 1:**
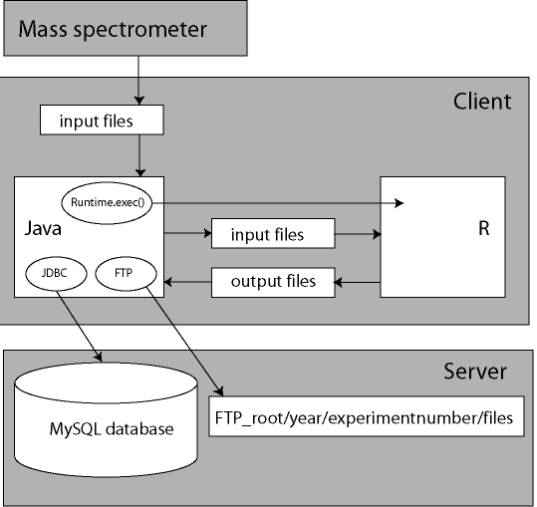
**System architecture**. The system architecture consists of a client JAVA code for fast processing of data while a MySQL database on the server contains all the MS metadata. An FTP service puts all the raw files and processed data on the server and client R is used for statistical analysis.

The MS analysis software architecture consists of 4 pillars, a Graphical User Interface (GUI) written in Java™ [28], a MySQL™ database [5], which contains all metadata, such as experiment numbers and sample codes, and a FTP (File Transport Protocol) server to store all raw MS fid files and processed data and fourth R. The software package R is used for statistical calculations [6]. Figure 1 gives a schematic overview of the architecture. The Java software components are developed and tested on the Eclipse™ platform [7]. The raw MS fid files can manually be selected by the Java GUI on the client and stored on a central FTP server. For calculations, the Java client retrieves the information in these files again. After processing of the data, the results of analysis are transported to the FTP server again. The FTP file storage is installed on a central server, and the information can be retrieved by different Java client workstations. However, for testing, the FTP service and MySQL database are both installed on the client workstation, with hostname *localhost*. Special Java archives (Jar's) have to be in the Java Virtual Machine's class path. The edtftpj-1.4.8.jar [8] provides an interface for programming the standard FTP commands in Java. The Java Database Connectivity (JDBC) driver mysql-connector-Java-3.1.6-bin.jar [5] gives an interface for SQL database access [9]. In this way, a communication between the Java client and the MySQL database or FTP service is established. There are several ways to set up an interface between Java and the statistical software package R [10]. Java's Runtime.exec() command is used in the database application. The advantage of applying this method is that it requires no other adaptations than a default installation of R. Lemkin et al[11] implemented the method in the Micro Array Explorer project. The Runtime.exec() command in Java can execute a Windows™ cmd.exe (command interpreter) batch file. The batch file, Rterm.bat, subsequently starts an Rterm™ process. The Rterm process has a file-based communication with Java (Figure 1). The Java client generates all R scripts and R input files. The name and path of the input and output files are defined in the generated R script. Java waits until Rterm has finished the job, and reads the output file(s). The Java application warns if Rterm is not installed in the default installation path on the client workstation.

### Database design

**Figure 2:**
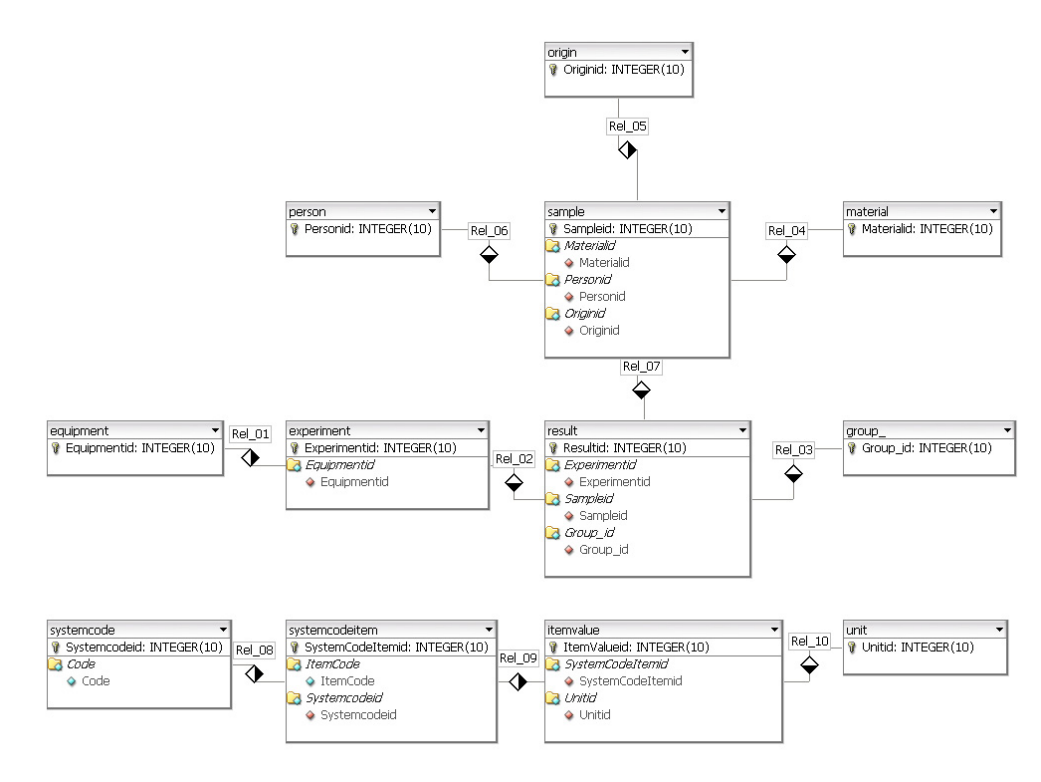
**Entity relationship diagram (ERD)**. Entity relationship diagram (ERD) of the MySQL proteomics database. The plot is created with dbdesigner4, a visual database designer for MySQL [12].

Figure 2 depicts the entity relationship diagram (ERD) of the MySQL database. The plot is created with dbdesigner4, a visual database designer for MySQL [12]. The database is kept at minimum size. The ERD distinguishes two sets of tables or entities. One set contains records with metadata of the MS measurements, namely equipment, experiment, result, sample, group_, person, material and origin. The other set consists of system tables. The records of the table result contain pointers to the MS files on the FTP server. These pointers are the filenames in the fields of these records, which hold also information about MALDI target plate spot positions. Each sample generates one or more mass spectra. Therefore, records in the table result keep the foreign key of the sample records. The database application selects the replicate spectra of each sample in order of the ascending *resultid *value of the result table. The reversed selection of replicate spectra is also studied by changing the order in descending *resultid *value of the result table. The samples have to be allocated to a certain group; control, breast cancer, or breast cancer with LM. The foreign key of the table group_ in the table result achieves indirectly a link between sample and group_. There is no direct link between the table sample and group_. In this way, samples can be allocated to different groups in different experiments. This gives more flexibility to the application, and avoids storage of redundant sample information. Information about the origin of a sample, for example *lumbar puncture*, can be stored in a table, as well as information about the material, *CSF*. A patient-id in records of the table person can link the MS results with other clinical data. Figure 2 shows also the second set of system supporting tables. These tables are named *systemcode, systemcodeitem, itemvalue*, and *unit*. The mass spectra can be internally calibrated with five omnipresent albumin peaks in CSF with a mass over charge (m/z) of 960.5631, 1000.6043, 1149.6156, 1511.8433 and 2045.0959Da [13,14]. These masses are stored in records of the table *itemvalue*. The series of these five calibration masses are named Albumin Masses in a record of the table *systemcodeitem*. The table *systemcode *offers the possibility to store more series of internal calibration masses than Albumin Masses in the system. The prostate cancer samples are internally calibrated with a different set of Albumin masses seen in most of the spectra, namely 1296.6594, 1511.7358, 1623.6973, 1639.8218, and 2044.9620Da.

### GUI components and functions

**Figure 3:**
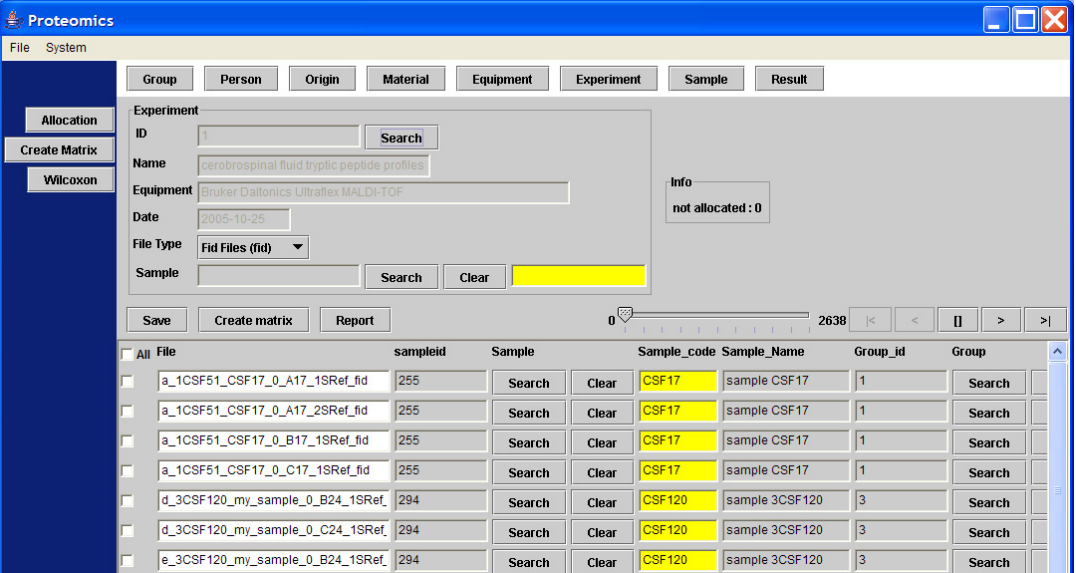
**Snapshot of the GUI to allocate the samples to different groups**. The allocation is achieved indirectly. Both samples and groups are linked to the mass spectra records in the table result, which contain the spot positions in their filenames.

**Figure 4:**
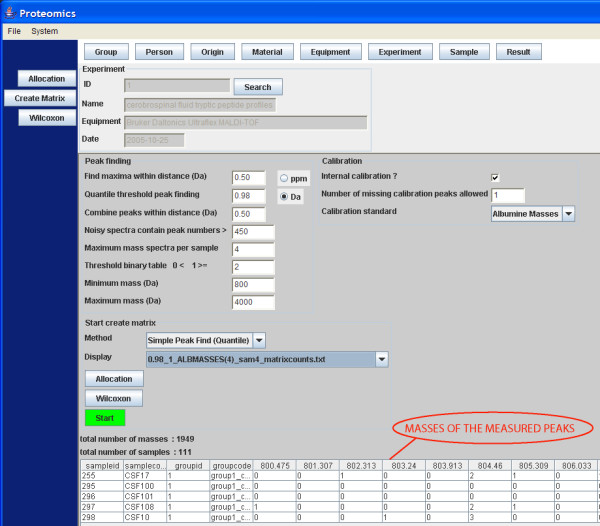
**Snapshot of the GUI to create one of the three matrices**. A matrix is created with the number of occurrences of masses in replicate spectra of different samples, a binary table with number of occurrences of masses above a specific threshold, and a matrix with the mean intensity of the present peaks in the mass spectra replicates. A small preview part of an example matrix with a total of 1949 peptide masses and 111 samples, with the number of occurrences of masses in replicate spectra of the samples is presented at the bottom of the GUI.

**Figure 5:**
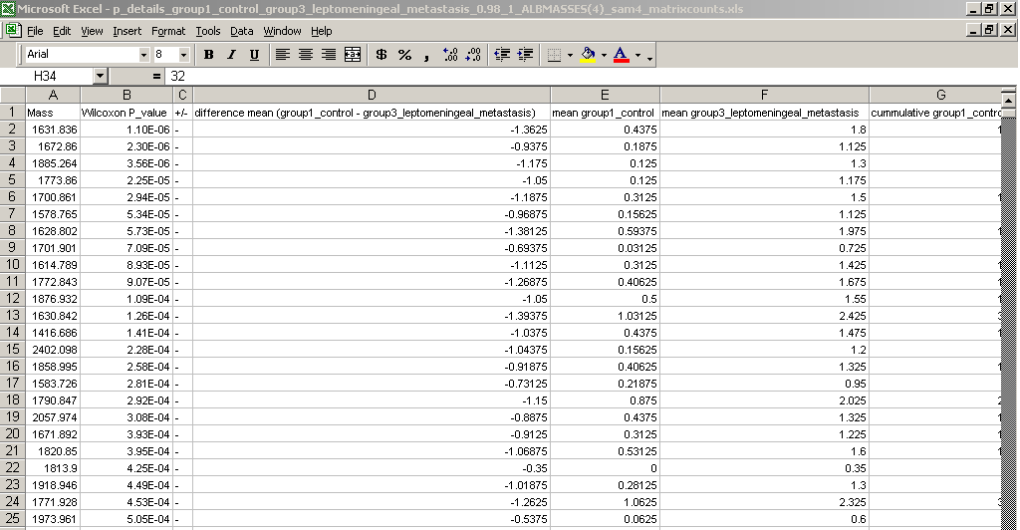
**Report of the Wilcoxon-Mann-Whitney p-values of the control group and the breast cancer with LM group**. An example of a detailed report of the Wilcoxon-Mann-Whitney p-values of the differentially expressed peptides. The report is stored on the FTP server, as well in the local document directory on the client workstation. The data are stored in a file with Comma Separated Value (CSV) format, which can be imported in Microsoft Excel™. The p-values of all peptide masses, as well positive (+) as negative (-) expressed between the groups are sorted in order of ascending p-values with the most significant discriminating peptide masses on top, with the lowest p-value. Negative (-) expressed means in this case that the mean peptides occurrences in the breast cancer with LM group are larger than in the control group.

**Figure 6:**
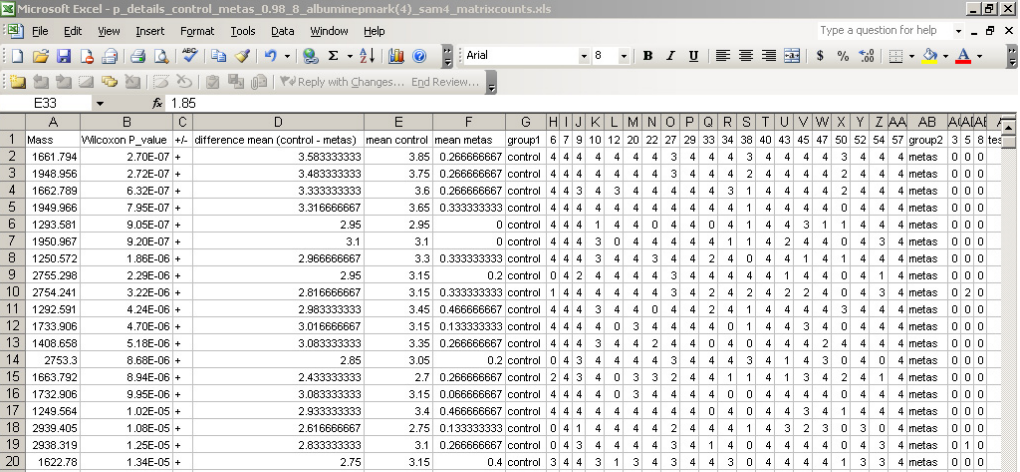
**Report of the Wilcoxon-Mann-Whitney p-values the control group and the end stage prostate cancer group**. An example of a detailed report of the Wilcoxon-Mann-Whitney p-values of the differentially expressed peptides. The report is stored on the FTP server, as well in the local document directory on the client workstation.

The software architecture contains the following GUI components and functions: 1) Import of the MS files from the (local) file system and to transport of these files to the FTP server; 2) search and selection of table records; 3) a screen to update or insert the records; 4) allocation the samples in different groups; 5) creating the profile matrix; and 6) performing the Wilcoxon-Mann-Whitney rank sum test on matrix values [15-17]. The GUI to select and import MS file to the FTP server is based on the Java's JFileChooser Class [9]. JFileChooser is a member of the Swing™ library for the GUI design. Most GUI components where build with this toolkit to keep the same look and feel throughout the application (except for the ugly JTextField), though SWT is getting more and more popular for these purposes, like the (SWT based) Eclipse IDE for development. One or more file(s) or even complete directories can be selected, and all files including subdirectories are transported to the FTP server location */ftp_root/year/experimentnumber*, for example C:\Inetpub\ftproot\2005\1\group2_f_2CSF20_0_B20_1SRef_fid. The combination of file type and the type of instrument determines how the data in the files should be processed. File types that can be imported into the system are at present binary fid and text files in ASCII format (American Standard Code for Information Interchange). This can be extended with any other file type. If the file type is fid, Bruker related acqu and acqus files, containing the calibration constants are also transported to the FTP server. The calibration constants have a totally different meaning for data measured with the TOF or FT-ICR technique. When the mass spectra are imported into the system, result records are created in the database. Each record of the table result refers to each mass spectrum, which is measured for a certain sample. For statistical analysis of the data, these result records have to be linked to samples and samples have to be placed in groups. The allocation module achieves this by constructing a link between the records of the result and sample table, and the result and group_ table, respectively (Figure 3). The field filename in a record of the result table holds the spot position the anchor chip, because it is part of the filename. Records in the table sample and table group_ hold the sample and group codes in their table fields. Table maintenance screens can add additional sample information, such as person, material, and origin. The matrix of number of occurrences of mass peaks in replicates of different samples allocated to different groups is created in another module (Figure 4). Three different matrices are produced simultaneously, one with the number of occurrences of masses in replicate spectra of different samples, a binary table with number of occurrences of masses above a specific threshold, and finally a matrix with the mean intensity of the present peaks in the mass spectra replicates. Figure 4 on bottom shows only a small fraction of a matrix with a total of 1949 peptide masses from 111 matrix samples, where the numbers of occurrences of the peptide masses in 4 replicate spectra of 5 matrix samples are visible. An example is the peptide mass of 804.46 Da, which is measured in a frequency of 2, 0, 0, 2 and 3 times for the 5 samples with code CSF17, CSF100, CSF101, CSF108, and CSF10, respectively (Figure 4). The complete matrix of all samples is stored in Comma Separated Value (CSV) format on the FTP server and in the local document root. The total matrix can be visualized by importing the table in the statistical package Spotfire. R's Wilcoxon-Mann-Whitney rank sum test is performed for each matrix peptide, based on the numbers of peptide mass occurrences per sample in different groups. The Wilcoxon-Mann-Whitney test discriminates the peptide masses between the groups with a probability value (p-value). The frequency distribution of the calculated p-values of the peptide masses in the matrix is presented in a histogram. A separate Wilcoxon-Mann-Whitney GUI generates this histogram and creates a list of the masses with corresponding p-values. In this screen, the test can be performed on matrices generated in different experiments and between different groups. The results of the Wilcoxon-Mann-Whitney rank sum test on a matrix are stored in a file with CSV format. The p-values of all peptide masses, as well positive (+) as negative (-) expressed between the groups are listed in this file. The file is stored on the FTP server and in the local document directory. Examples of these p-value listings are given for the breast cancer with LM group against the control group in Figure 5, and for the prostate cancer group in end stage disease against another control group in Figure 6. Peptide masses with the lowest p-values < 0.01 are used to search in the Human MSDB database for proteins based on calculated peptide masses, using MASCOT or are selected for MS/MS sequencing options in the mass spectrometer.

### Calibration constants

A small storage size of the files on the FTP server is guarantied, due to the fid format of MS spectra, a byte array of 92000 channel intensities. The TOF, time, can be calculated from the MS channel number, i, in the fid files by

*time*_*i *_= *DELAY *+ (*i*·*DW*) *i *= 1,2,...,92000       (1)

The values of the constants DW (dwell time) and DELAY are stored in the acqus and acqu files, which are also transported to the FTP server. Other important values are those of the ML1, ML2 and ML3 calibration constants in the acqus files, which are used to calculate the peptide masses from the TOF. Theoretically, the square root of the mass over charge, mz
 MathType@MTEF@5@5@+=feaafiart1ev1aaatCvAUfKttLearuWrP9MDH5MBPbIqV92AaeXatLxBI9gBaebbnrfifHhDYfgasaacH8akY=wiFfYdH8Gipec8Eeeu0xXdbba9frFj0=OqFfea0dXdd9vqai=hGuQ8kuc9pgc9s8qqaq=dirpe0xb9q8qiLsFr0=vr0=vr0dc8meaabaqaciaacaGaaeqabaqabeGadaaakeaadaGcaaqaamaalaaabaGaemyBa0gabaGaemOEaOhaaaWcbeaaaaa@2FB7@, is proportional with the TOF, time.

0=A⋅(mzi)2+B⋅mzi+C(timei)     (2)
 MathType@MTEF@5@5@+=feaafiart1ev1aaatCvAUfKttLearuWrP9MDH5MBPbIqV92AaeXatLxBI9gBaebbnrfifHhDYfgasaacH8akY=wiFfYdH8Gipec8Eeeu0xXdbba9frFj0=OqFfea0dXdd9vqai=hGuQ8kuc9pgc9s8qqaq=dirpe0xb9q8qiLsFr0=vr0=vr0dc8meaabaqaciaacaGaaeqabaqabeGadaaakeaacqaIWaamcqGH9aqpcqWGbbqqcqGHflY1daqadiqaamaakaaabaWaaSaaaeaacqWGTbqBaeaacqWG6bGEdaWgaaWcbaGaemyAaKgabeaaaaaabeaaaOGaayjkaiaawMcaamaaCaaaleqabaGaeGOmaidaaOGaey4kaSIaemOqaiKaeyyXIC9aaOaaaeaadaWcaaqaaiabd2gaTbqaaiabdQha6naaBaaaleaacqWGPbqAaeqaaaaaaeqaaOGaey4kaSIaem4qamKaeiikaGIaemiDaqNaemyAaKMaemyBa0Maemyzau2aaSbaaSqaaiabdMgaPbqabaGccqGGPaqkcaWLjaGaaCzcamaabmGabaGaeGOmaidacaGLOaGaayzkaaaaaa@507B@

Therefore, the value of constant B is about 40.000 times larger than the value of constant A, where *A *= *ML*3, *B *= 1012ML1
 MathType@MTEF@5@5@+=feaafiart1ev1aaatCvAUfKttLearuWrP9MDH5MBPbIqV92AaeXatLxBI9gBaebbnrfifHhDYfgasaacH8akY=wiFfYdH8Gipec8Eeeu0xXdbba9frFj0=OqFfea0dXdd9vqai=hGuQ8kuc9pgc9s8qqaq=dirpe0xb9q8qiLsFr0=vr0=vr0dc8meaabaqaciaacaGaaeqabaqabeGadaaakeaadaGcaaqaamaalaaabaGaeGymaeJaeGimaaZaaWbaaSqabeaacqaIXaqmcqaIYaGmaaaakeaacqWGnbqtcqWGmbatcqaIXaqmaaaaleqaaaaa@3402@, and *C*(*time*_*i*_) = (*ML*2 – *time*_*i*_). The mass over charge is

mzi=(−B+B2−4⋅A⋅C(timei)2A)2     (3)
 MathType@MTEF@5@5@+=feaafiart1ev1aaatCvAUfKttLearuWrP9MDH5MBPbIqV92AaeXatLxBI9gBaebbnrfifHhDYfgasaacH8akY=wiFfYdH8Gipec8Eeeu0xXdbba9frFj0=OqFfea0dXdd9vqai=hGuQ8kuc9pgc9s8qqaq=dirpe0xb9q8qiLsFr0=vr0=vr0dc8meaabaqaciaacaGaaeqabaqabeGadaaakeaadaWcaaqaaiabd2gaTbqaaiabdQha6naaBaaaleaacqWGPbqAaeqaaaaakiabg2da9maabmGabaWaaSaaaeaacqGHsislcqWGcbGqcqGHRaWkdaGcaaqaaiabdkeacnaaCaaaleqabaGaeGOmaidaaOGaeyOeI0IaeGinaqJaeyyXICTaemyqaeKaeyyXICTaem4qamKaeiikaGIaemiDaqNaemyAaKMaemyBa0Maemyzau2aaSbaaSqaaiabdMgaPbqabaGccqGGPaqkaSqabaaakeaacqaIYaGmcqWGbbqqaaaacaGLOaGaayzkaaWaaWbaaSqabeaacqaIYaGmaaGccaWLjaGaaCzcamaabmGabaGaeG4mamdacaGLOaGaayzkaaaaaa@5144@

### Peak finding

A peak list consists of mass over charge (m/z), channel number i, and intensity. It is constructed from the data in the raw fid files. A histogram of the number of channels with a specific intensity can be constructed. The integral under the distribution curve represents the amount of 92000 instrument channels. From this distribution curve, the R quantile function calculates an intensity threshold, where the probability is 98 % to find channels with a lower intensity. The effect of changing R quantile percentages between 97 and 99 % in the create matrix GUI (Figure 4) is examined. The MS peaks are expected to be in the channels numbers, i, with intensity higher than this threshold, namely in the range of the 3 % highest intensities. The peak finding algorithm determines the highest channel intensity within a certain mass over charge (m/z) window, for example 0.5 Da at both sides. A second condition is that this local maximum intensity must be above the quantile threshold intensity. Noise spectra do not contain real peaks with a high intensity flanks. As a consequence, many noise peaks are above the quantile threshold. Peak lists with too many peak masses above an arbitrary number of 450 fall off, because a large part of these peak positions are probably noise peaks.

### Internal calibration

Internal calibration is necessary to align all the spectra in the matrix. There are several methods reported to align mass spectra datasets. The alignment algorithms of Wong et al. [18] and Jeffries [19] have in common that they use special reference masses or peaks between the spectra. Wong et al. [18] have developed an algorithm written in C^++ ^where spectral data points are added or deleted in regions with a low intensity, in order to a shift peaks. This algorithm has a slight effect on the shape of the peaks. However, the signals in MS are presented by peaks and not by the regions of minimal intensity. Jeffries [19] compares peaks lists generated from mass spectra. He uses R's smooth spline function to correct measured masses with help of reference calibrate masses. A smooth spline function, f_λ_, is drawn through the ratio of measured over real mass on the y-axis against the measured mass of the calibrate peaks on the x-axis, which results in a factor close to 1. Division of the measured masses by the calculated function f_λ_; interpolates all data points. Theoretically, a cubic spline function needs to pass through all of the calibrate data points. This results in a lot of curvature. A smooth spline is a compromise; where the function may deviate from calibrate data points within a certain limit, due to a factor λ, which diminishes the amount of slope. The amount of slope is expressed by the integrating the square of the second derivative of the spline function [19]. Another alignment algorithm assumes no knowledge of peaks in common [20,21]. This method considers the shape of the spectra, and aims to minimize the phase differences between the spectra. This process is named dynamic time warping. It is however easier to calibrate the channel numbers of the MALDI-TOF equipment against known masses, since the square root of mass over charge is theoretically proportional to the time. This dependency can be fit with a polynomial function. The masses in the peak list are internally calibrated, using the at least 4 of the 5 omnipresent albumin masses. The channel numbers in the peak list, with corresponding masses, which are the closest with a window of 0.5 Da to one of the albumin masses, are determined. Peak lists without the required number of albumin masses fall off. The channel numbers, i, and corresponding albumin masses, mzi
 MathType@MTEF@5@5@+=feaafiart1ev1aaatCvAUfKttLearuWrP9MDH5MBPbIqV92AaeXatLxBI9gBaebbnrfifHhDYfgasaacH8akY=wiFfYdH8Gipec8Eeeu0xXdbba9frFj0=OqFfea0dXdd9vqai=hGuQ8kuc9pgc9s8qqaq=dirpe0xb9q8qiLsFr0=vr0=vr0dc8meaabaqaciaacaGaaeqabaqabeGadaaakeaadaWcaaqaaiabd2gaTbqaaiabdQha6naaBaaaleaacqWGPbqAaeqaaaaaaaa@3123@, are fit in a second-degree polynomial function

mzi=a[1]⋅i2+a[2]⋅i+a[0]     (4)
 MathType@MTEF@5@5@+=feaafiart1ev1aaatCvAUfKttLearuWrP9MDH5MBPbIqV92AaeXatLxBI9gBaebbnrfifHhDYfgasaacH8akY=wiFfYdH8Gipec8Eeeu0xXdbba9frFj0=OqFfea0dXdd9vqai=hGuQ8kuc9pgc9s8qqaq=dirpe0xb9q8qiLsFr0=vr0=vr0dc8meaabaqaciaacaGaaeqabaqabeGadaaakeaadaWcaaqaaiabd2gaTbqaaiabdQha6naaBaaaleaacqWGPbqAaeqaaaaakiabg2da9iabdggaHnaadmGabaGaeGymaedacaGLBbGaayzxaaGaeyyXICTaemyAaK2aaWbaaSqabeaacqaIYaGmaaGccqGHRaWkcqWGHbqydaWadiqaaiabikdaYaGaay5waiaaw2faaiabgwSixlabdMgaPjabgUcaRiabdggaHnaadmGabaGaeGimaadacaGLBbGaayzxaaGaaCzcaiaaxMaadaqadiqaaiabisda0aGaayjkaiaawMcaaaaa@4CBC@

The coefficients, a [0], a[1], and a[2] are calculated with R's linear model (lm) function where *y *= mzi
 MathType@MTEF@5@5@+=feaafiart1ev1aaatCvAUfKttLearuWrP9MDH5MBPbIqV92AaeXatLxBI9gBaebbnrfifHhDYfgasaacH8akY=wiFfYdH8Gipec8Eeeu0xXdbba9frFj0=OqFfea0dXdd9vqai=hGuQ8kuc9pgc9s8qqaq=dirpe0xb9q8qiLsFr0=vr0=vr0dc8meaabaqaciaacaGaaeqabaqabeGadaaakeaadaWcaaqaaiabd2gaTbqaaiabdQha6naaBaaaleaacqWGPbqAaeqaaaaaaaa@3123@, *x *= *i*, and *a *is the array of a [0], a[1], and a[2]

*ft*3 ← *lm*(*y *~*I*(*x*^2) + *x*)       (5)

*a *← *coeff *(*ft*3)       (6)

All peptide masses in the peak list are recalculated, using these coefficients and the polynomial function.

### Data reduction

**Figure 7:**
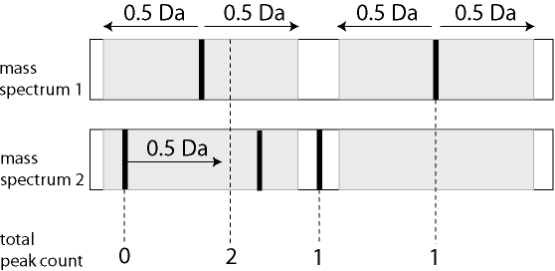
**Data reduction**. Data reduction by finding peak maxima and combining the measured peptide masses in the different spectra. Occurrences of masses with a window of 0.5 Da are summed, and the average value of the mass is calculated (dashed line). The occurrence of only one peptide in the second spectrum is summed in the mass window of the first spectrum. The second peak of spectrum 2 and not the first one is combined with the first peak of spectrum 1, since it has the closest distance in mass (Da) with the first peak of spectrum 1. Not previously registered masses in the first spectrum are added to the list. The clustering continues iteratively through all mass spectra of the samples in the matrix.

Last step is the creation of the profile matrix, which consists of peptide masses in all spectra of the samples in the columns against the occurrences in replicate spectra of the samples in the rows. The matrix is the input file for the Wilcox-Mann-Whitney test, but can also be input for other statistical packages, like Spotfire. The matrix is stored on the FTP server, as well as in the local document directory. Figure 7 schematically shows the clustering of two spectra in the matrix. Within a mass window of 0.5 Da at both sides of a peptide mass in the first spectrum, the occurrence of at least one peptide mass in the second spectrum is investigated, closest in distance to the peak in the first spectrum. If that is the case, the average mass of both peptide masses is calculated (dashed line in Figure 7), and the number of occurrences in both spectra summed. For each mass spectrum, only one peptide occurrence, 1 or 0, is summed for each mass window. If a peptide mass is present in the second spectrum, but not in the first spectrum it is added to the mass list. The average intensity of the present masses is also stored in a separate matrix. The clustering continues iteratively though all spectra of the samples in the matrix. All averages are calculated at the end of the clustering routine. In the database application, the clustering of selected spectra is in the order of ascending *groupid*, ascending *sampleid*, and ascending *resultid *values in records of the table result, which are pointers to the files on the FTP server. The effect of reversed clustering is studied by changing the order in descending *groupid*, descending *sampleid*, and descending *resultid *values of the table result.

## Results

**Figure 8:**
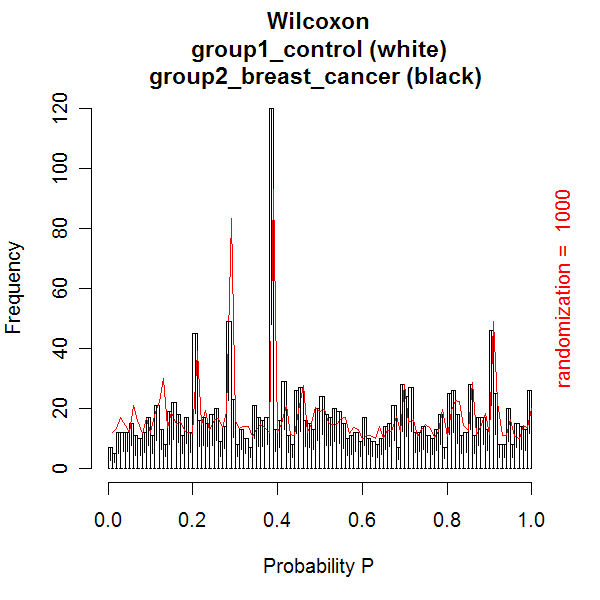
**Histogram of the number of peptide masses with Wilcoxon-Mann-Whitney probability p-values of the control versus breast cancer group**. All 1949 identified peptide masses in the matrix are presented as a function of the Wilcoxon-Mann-Whitney probability p-value. The Wilcoxon-Mann-Whitney p-value is calculated for each peptide mass based on its occurrences in replicate samples in each group. The white bars represent the frequency of the peptide masses with a p-value in the control group, while the black bars represent the frequency of the peptide masses with a p-value in the breast cancer group.

**Figure 9:**
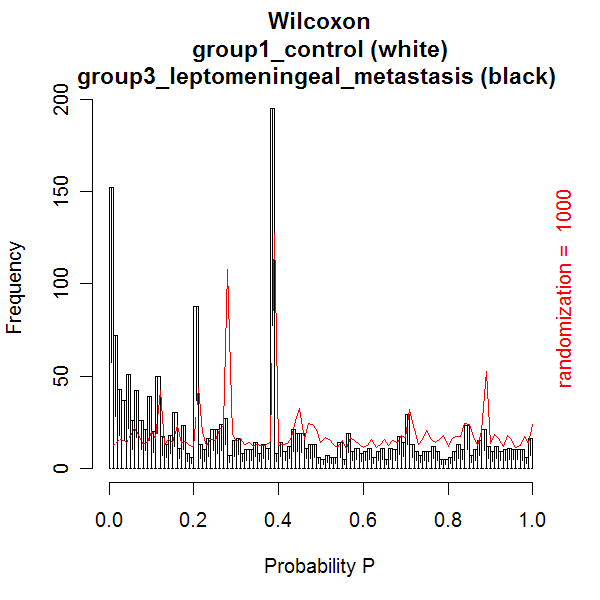
**Histogram of the number of peptide masses with Wilcoxon-Mann-Whitney probability p-values of the control versus the breast cancer with LM group**. All 1949 identified peptide masses in the matrix are presented as a function of the Wilcoxon-Mann-Whitney probability p-value. The white bars represent the frequency of the peptide masses with a p-value in the control group, while the black bars represent the frequency of the peptide masses with a p-value in the breast cancer with LM group. 152 peptide masses are significantly differentially expressed in the breast cancer with LM group (p-value less than 0.01, ranging from 1.1 * 10^-6 ^to 9.8 * 10^-3^).

**Figure 10:**
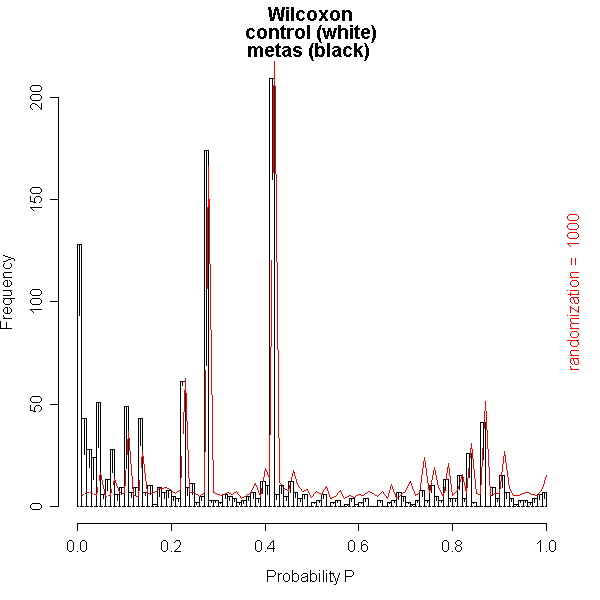
**Histogram of the number of peptide masses with Wilcoxon-Mann-Whitney probability p-values of the control versus the end stage prostate cancer group**. All 1354 identified peptide masses in the matrix are presented as a function of the Wilcoxon-Mann-Whitney probability p-value. The white bars represent the frequency of peptide masses in the control group with a specific p-value, while the black bars represent the frequency of peptide masses with a p-value in the prostate cancer in end stage disease group. 128 peptide masses have a p value of less than 0.01, ranging from 2.7 * 10^-7 ^to 9.9 * 10^-3^.

The first data set with CSF mass spectra was previously reported by Dekker and co-workers [3,4]. The optimal quantile percentage of 98% in peptide profiling [3,4] is confirmed in our database application. At a relative low quantile percentage of 97%, close to the noise level, many spectra are rejected due to a large amount of noise peaks with numbers above the threshold of 450. This result in a relatively low number of matrix samples due to too low replicate numbers. The matrix consists of peptide masses present in all spectra of the samples in columns against the occurrences in replicate spectra of the samples in the rows. At a high quantile percentage of 99%, internal calibration is not possible for many spectra, because albumin peak intensities are below the quantile threshold. Again many samples have too low replicate numbers, resulting in a relatively low number of matrix samples. An effect of reversed selection of replicate spectra of samples is expected and measured, when replicate numbers are smaller than the (dataset dependent) value of 7, since the replicate spectra contribute a different part of the total identified peptide masses. At replicate numbers above this value of 7, no effect of changing the order of selecting replicates is expected. No effect of reversed clustering of the selected spectra is measured on the number of samples and peptide masses in the matrix (Figure 7). The Wilcoxon-Mann-Whitney test is best performed on a matrix with the most samples and peptide masses, generated at a quantile percentage of 98%. Figures 8, 9, 10 show examples of the distributions of the frequency of all the peptides in the matrix as a function of the Wilcoxon-Mann-Whitney p-value, a) of the control group versus the group with breast cancer (Figure 8), b) of the control group versus the group of breast cancer with LM (Figure 9), and c) of another control group versus end stage prostate (Figure 10). There are some differences in shape of the Wilcoxon-Mann-Whitney probability p-value frequency distributions presented in Figures 8, 9, 10 with those presented earlier [3], since we used a mass range of 800 to 4000 (previously 300–3000 [3]) and we did not leave out peptide masses, which are measured in less than 5 % of the spectra, which results in sharp peaks at p-values of 0.20, 0.28, and 0.38. The frequency histograms of the Wilcoxon-Mann-Whitney p-values of both comparisons a) and b) of the breast cancer patients clearly display a different shape. All peptides are equally distributed over all p-values for the control group versus the group with breast cancer in Figure 8. A line is drawn in the plot, which represents the frequency of p-values of peptides after a 1000 times scrambling of the samples over the two groups in the matrix. This random distribution is about equal to the measured pattern of the control group versus the group with breast cancer. Some sharp frequency peaks are calculated at p-values of 0.20, 0.28, and 0.38. The p-values of 0.28 and 0.38 are calculated when a peptide mass is measured once in a spectrum of one sample in the control group (smallest group) or in the group with breast cancer (largest group) of the matrix. This occurs quite often, as can be seen by the relatively high frequency in the histogram at these p-values. Randomization of the sample with this single peptide peak over the groups hardly changes the frequency of these p-values. This is confirmed by the maximum values of the solid randomization line (Figure 8). The p-value of 0.20 corresponds to peptide masses found in total two times in two different samples in one group. As expected, by randomization, the frequency of this p-value decreases in favor of the p-values of 0.28 or 0.38, since the two samples can be allocated to two groups. The solid line displays a small decrease of the frequency at p-values 0.20 and larger increase of the frequency at p-value 0.28. The frequency of p-values shows a small decline below 0.01, which means that there is a small amount of differentially expressed peptides. The shape of the frequency distribution of Wilcoxon-Mann-Whitney p-values for the peptide masses is remarkably different when we compare the CSF peptide profiles from the control group with the profiles from the group of breast cancer with LM (Figure 9). Again sharp peaks in frequency are observed at p-values of 0.20 and 0.38, and a smaller peak at 0.28. However, the histogram shows also sharp increase of the frequency of peptides with p-values below 0.01. This indicates that there are significantly differentially expressed peptides present in the CSF from the LM breast cancer patients. A total of 1949 peptide masses are identified in the 45 control and 54 LM breast cancer samples. Of these, 152 peptide masses have a p-value < 0.01, ranging from 1.1 * 10^-6 ^to 9.8 * 10^-3^. A similar pattern of the Wilcoxon-Mann-Whitney p-value distribution is seen in Figure 10, when the end stage prostate cancer samples are compared with those of the control group. Again three maxima are observed at p-values of 0.23 for in total two peptide mass occurrences in separate samples in one group, 0.27 for one occurrence in one sample in the smaller metastasis group, and 0.42 for one occurrence in one sample of the larger control group. Again an increasing number of peptide masses with p-values < 0.01 are seen in the histogram. A total of 1354 peptide masses are identified in the 20 control samples and 15 end stage prostate cancer samples. Of these, 128 peptide masses have a p-value < 0.01, ranging from 2.7 * 10^-7 ^to 9.9 * 10^-3^.

## Discussion

The database application can clearly distinguish the MALDI-TOF peptide profiles between different patient and control groups. It can determine differences in the frequency and intensities of peptide masses in spectra from both groups. A strong feature of the here described architecture is that it can process different MS file formats, such as peak lists, MALDI-TOF and FT-ICR binary files from various manufactures in the same manner. More important are speed and memory usage by the client workstation. Peptide profile matrices have to be created in reasonable time. When dealing with large quantity of data, the Java application will easily run into out of memory errors with default settings of the JVM. Very important to use limit and offset strategies in MySQL queries to fetch no more than a buffered amount of 5000 table records each time when displaying them in the GUI. A specific MALDI-TOF MS matrix of 111 samples and 1949 masses (Figure 4) has 216339 matrix fields and a CSV file size of 444 Kbytes. Three matrices, peptide mass occurrences, intensity, and binary of this size can be simultaneously built in the Java Virtual Machine's (JVM) allocated memory. However, a typical FTMS matrix with 374 samples and 10651 discriminated masses has an 18 times larger number of 3983474 matrix fields and an 18 times larger CSV file size of 7.9 Mbytes. It is impossible to build three matrices of these size simultaneously in the Java's memory space. These files have to be built in the user document root as a FileOutputStream and transported to the FTP server.

### Architecture

It is also possible to use the SJava package [22] to set up an interface between Java and the statistical software package R. It can be used to invoke Java methods and create Java objects by R commands. This is, however, the opposite of our approach, where R is called from Java. Another approach is to access R by a TCP-IP (Transmission Control Protocol-Internet Protocol) connection, using the service Rserve™. A disadvantage of using this method is that the Rserve has to be started explicitly by the operating system out of the Java application before running any R script from Java. It would be possible to make additional java classes for statistical routines, such as the Wilcoxon-Mann-Whitney test. Indeed for this one test it would be more logical to add it directly to the Java code. However, the usage of R goes beyond just the Wilcoxon-Mann-Whitney test, which is not being claimed to be the full analysis. The Wilcoxon-Mann-Whitney test is an example of a univariate test that is an important first step. In R, it is possible to switch to other univariate tests and most importantly multivariate analysis, such as hierarchical clustering in two dimensions (where Spotfire™ fails with very big matrices). In addition, R can be used for the peak finding algorithms (quantile calculations, baseline and noise level determination, etc.) which have the advantage that these algorithms are well tested and optimized for speed. The architecture allows the analysis to be extended to clustering, the building of multivariate classifiers, etc. (techniques we have already used in our previous paper [3]). This will be an important point to focus on in the future. A reason to implement a 2-tier architecture, thick client and database server, is to have an attractive Java GUI than less advanced interface and Java script in a web browser. It's possible to monitor preprocessing of the MS spectra with a progress bar. Another possibility is to convert instrument specific file types to uniform mzXML file format and display spectra with a Java mzXML viewer [29]. A 3-tier architecture with presentation layer (web-browser), business logic provided by an application server, and a database server is more difficult to implement. For example, the file interface of Java with the software package R is more difficult than in the 2-tier architecture. In the 2-tier situation every user has its own file repository on the local machine. In a 3-tier, special precautions have to be taken to prevent time-out errors and performance issues, applying distributed computing in a grid. For example, an FT-ICR MS peptide profile matrix of 10651 discriminated masses and 374 samples with at least 3 peptide occurrences per mass has a size of 7887 Kbytes and is produced in no less time than 12 h.

### Pre-processing

More advanced techniques such as Fourier transform ion cyclotron resonance (FT-ICR) MS and offline nano LC-MALDI (Liquid Chromatography) in combination with FT-ICR measure accurate masses in the 0.5 to 1 ppm range. Furthermore, the higher resolution of FT-ICR MS prevents the clustering of peaks of different peptides. These techniques allow the identification of proteins from peptide masses by either peptide mapping or peptide sequencing. The database application can be adapted to handle the mass spectra of these experiments due to its modular architecture. The type of equipment, in combination with type of imported spectra will determine the handling of raw data, such as calibration and peak finding algorithms. In order to transform the spectra from the time domain to the frequency domain [23], an extra Fast Fourier Transformation (FFT) step to handle raw data of FT-ICR experiments is at present under construction. The peptide masses can subsequently be calculated from the cyclotron frequency. It is also possible to apply a de-isotope algorithm on the peptide masses due to the higher resolution and mass accuracy of FT-ICR. Peak centroiding will be implemented, which calculates the real mass of the peak maximum, weighted by the intensity of the points surrounding the local maximum[24].

### Database

The database is stripped to its essentials and contains all the necessary fields for preprocessing while most of the input parameters are stored in the matrices filenames. For example, the database design does not contain an authentication database for encrypted password storage and management of user accounts. Other tables that are not included are audit trail and action logging tables found in modern LIMS. Details of the database design are presented in the proteomics.txt create table script (added as a supplementary file). The database tables contain the necessary (on delete) table triggers, which ensure database integrity. The database design allows the comparison of large quantity of mass spectra. The table result offers the possibility to store retention times and to group sequential mass spectra from one sample in a LC-MS experiment. An improved peak finding algorithm based on signal to noise levels is under construction [25]. An extra table with calculated peptide masses of expected proteins from MS-MS experiments can be added to the database, which will make a direct analysis of differentially expressed proteins possible.

### FTP

The use of an ordinary FTP server in a university environment is a security risk that cannot be underestimated. On the other hand, FTP is a standard that is accepted and widely accessible across every network and operating system. First of all, precautions have to be taken with setup and configuration of an FTP server as described e.g. by Ray Zadjmool [26]. The architecture disables anonymous access. However it not possible to register user accounts and connection is made by one root account. Users and IP addresses have to be logged as well as success of fail of account logon events. Account should be locked after several failed login attempts. Access to the FTP directory should be regulated using access control list (ACL) restrictions across Windows NT File System (NTFS) permissions. Disk quota should be enabled to limit the amount of disk space of a user, to prevent becoming a media file share place for hackers. IP address restriction should be set equal to the range of Hospital or University IP addresses. The user passwords must meet complexity requirements. However, FTP servers can only handle usernames and passwords in plain text, which can easily be intercepted by password sniffers. Sensitive data and login information can be encrypted for total security using FTPS or SFTP, which solves the problem of insecure FTP. FTPS (FTP over SSL) uses a Secure Socket program Layer (SSL) located between the FTP and Transport Control Protocol (TCP) layers. FTPS has the encryption capabilities of SSL with the advanced features of FTP. Unfortunately, Enterprise Distributed Technologies [8] provides only a commercial Java library, which supports FTPS and SFTP. An open source Java secure shell version 2 (SSH-2) library, jsch-0.1.28.jar, that supports SFTP (FTP over SSH) is provided by JCraft [27].

### Bonferroni

In the Bonferroni approach to n independent tests, the overall change β to make an error of type 1 is the product of the individual errors β'.

β = β'_1 _* β'_2 _* β'_3 _......... β'_n _= (1-p')^n ^= 1 - p       (7)

According to the binominal theorem, for small values of p'

(1−p′)n=1−np′+n(n−1)2(−p′)2+....+(nk)(−p′)k+...+n(−p′)n−1+(−p′)n≈1−np′     (8)
 MathType@MTEF@5@5@+=feaafiart1ev1aaatCvAUfKttLearuWrP9MDH5MBPbIqV92AaeXatLxBI9gBaebbnrfifHhDYfgasaacH8akY=wiFfYdH8Gipec8Eeeu0xXdbba9frFj0=OqFfea0dXdd9vqai=hGuQ8kuc9pgc9s8qqaq=dirpe0xb9q8qiLsFr0=vr0=vr0dc8meaabaqaciaacaGaaeqabaqabeGadaaakeaadaqadiqaaiabigdaXiabgkHiTiqbdchaWzaafaaacaGLOaGaayzkaaWaaWbaaSqabeaacqWGUbGBaaGccqGH9aqpcqaIXaqmcqGHsislcqWGUbGBcuWGWbaCgaqbaiabgUcaRmaalaaabaGaemOBa42aaeWaceaacqWGUbGBcqGHsislcqaIXaqmaiaawIcacaGLPaaaaeaacqaIYaGmaaWaaeWaceaacqGHsislcuWGWbaCgaqbaaGaayjkaiaawMcaamaaCaaaleqabaGaeGOmaidaaOGaey4kaSIaeiOla4IaeiOla4IaeiOla4IaeiOla4Iaey4kaSYaaeWaceaafaqabeGabaaabaGaemOBa4gabaGaem4AaSgaaaGaayjkaiaawMcaamaabmGabaGaeyOeI0IafmiCaaNbauaaaiaawIcacaGLPaaadaahaaWcbeqaaiabdUgaRbaakiabgUcaRiabc6caUiabc6caUiabc6caUiabgUcaRiabd6gaUnaabmGabaGaeyOeI0IafmiCaaNbauaaaiaawIcacaGLPaaadaahaaWcbeqaaiabd6gaUjabgkHiTiabigdaXaaakiabgUcaRmaabmGabaGaeyOeI0IafmiCaaNbauaaaiaawIcacaGLPaaadaahaaWcbeqaaiabd6gaUbaakiabgIKi7kabigdaXiabgkHiTiabd6gaUjqbdchaWzaafaGaaCzcaiaaxMaadaqadiqaaiabiIda4aGaayjkaiaawMcaaaaa@731C@

These equations show that the overall p-value threshold, p, should be divided by n to obtain the significance level, p' of the individual tests.

p′=pn     (9)
 MathType@MTEF@5@5@+=feaafiart1ev1aaatCvAUfKttLearuWrP9MDH5MBPbIqV92AaeXatLxBI9gBaebbnrfifHhDYfgasaacH8akY=wiFfYdH8Gipec8Eeeu0xXdbba9frFj0=OqFfea0dXdd9vqai=hGuQ8kuc9pgc9s8qqaq=dirpe0xb9q8qiLsFr0=vr0=vr0dc8meaabaqaciaacaGaaeqabaqabeGadaaakeaacuWGWbaCgaqbaiabg2da9maalaaabaGaemiCaahabaGaemOBa4gaaiaaxMaacaWLjaWaaeWaceaacqaI5aqoaiaawIcacaGLPaaaaaa@35D4@

In both the CSF and prostate cancer datasets some tests satisfy the Bonferroni multiple test approach, for example 1.1 * 10–6 < 0.01/1949 and 2.7 * 10–7 < 0.01/1354. The Bonferroni approach may not be ideally suited for this type of data as the presence of individual peptide peaks may be correlated, since they can be isotopes of the same peptide or peptides from the same protein. Rather than lowering the p-value threshold in a Bonferroni approach, the complete p-value distribution (and a randomization method to check the expected distribution) is shown. The numbers are explicitly supplied, because the plot does not specify the exact p-values lower than 0.01.

## Conclusion

A new software architecture is presented which can analyze high throughput MS data from MALDI-TOF MS measurements in a efficient way. Results of the analysis are stored in a centralized relational database and FTP server. Meta data of the experiment and samples can be stored as well, and can be used to link the results to clinical data or data from other types of experiments. The database application generates a matrix with the frequency of masses in replicate spectra from different samples, a binary table with the frequency of masses above a specific threshold, and a matrix with the mean intensity of the present peaks in the mass spectra replicates. The matrix, which is stored on the FTP server and in the local document directory, can be imported in statistical packages or in (commercial) analysis software such as Spotfire. Statistical analysis of two test datasets by the Wilcoxon-Mann-Whitney test in R clearly distinguishes the peptide-profiles of patient body fluids from those of controls. Finally, the modular architecture of the application makes it possible to also handle data from FT-ICR experiments.

## Availability and requirements

Java source code, create table script and installation instructions are added as additional files [see Additional file 1, 2, 3, 4, 5 and 6].

## Abbreviations

ACL: Access Control List

ASCII: American Standard Code for Information Interchange

CSF: Cerebrospinal Fluid

CSV: Comma Separated Value

DW: Dwell (time)

ERD: Entity Relationship Diagram

FFT: Fast Fourier Transformation

FID: Free Induction Decay

FT-ICR: Fourier Transform Ion Cyclotron Resonance

FTP: File Transport Protocol

GUI: Graphical User Interface

IP: Internet Protocol

JAR: Java Archive

JVM: Java Virtual Machine

JDBC: Java Database Connectivity

LC: Liquid Chromatography

LIMS: Laboratory Information Management System

LM: Leptomeningeal Metastasis

MALDI-TOF: Matrix Assisted Laser Desorption Ionization

MS: Mass Spectrometry

mzXML: mass over charge eXtensible Markup Language

NTFS: Windows NT File System

SSH-2: Secure Shell version 2

SSL: Secure Socket program Layer

SQL: Structured Query Language

SWT: Standard Widget Toolkit

TCP: Transmission Control Protocol

## Authors' contributions

MKT generated and tested the Java code and GUI. LJD and ALCTR performed sample preparations and MS experiments, and IS programmed the R routines. All authors read and agreed with the manuscript

## Supplementary Material

Additional file 1 Installation instructions in README. Click here for file

Additional file 2 Create table script proteomics. Click here for file

Additional file 3 Java Source code as a proteomics. Click here for file

Additional file 4 Bruker Daltonics data for testing the application (fid files) Click here for file

Additional file 5 Detailed Wilcoxon-Mann-Whitney p-values listing of the breast cancer with LM patients against a control group Click here for file

Additional file 6 Detailed Wilcoxon-Mann-Whitney p-values listing of the end stage prostate cancer patients against a control group Click here for file
